# *Lycium barbarum* Polysaccharides Prevent Memory and Neurogenesis Impairments in Scopolamine-Treated Rats

**DOI:** 10.1371/journal.pone.0088076

**Published:** 2014-02-05

**Authors:** Weiwei Chen, Xiang Cheng, Jinzhong Chen, Xin Yi, Dekang Nie, Xiaohui Sun, Jianbing Qin, Meiling Tian, Guohua Jin, Xinhua Zhang

**Affiliations:** 1 Department of Anatomy, Nantong University, Nantong, Jiangsu, China; 2 Department of Radiotherapy, The 3rd People’s Hospital of Yancheng, The Affiliated Yancheng Hospital of Southeast University, Yancheng, Jiangsu, China; 3 Department of Neurosurgery, Affiliated Hospital of Nantong University, Nantong, Jiangsu, China; 4 Cardiovascular Department, Rehabilitation Hospital of Agings, Nantong, Jiangsu, China; Hospital General Dr. Manuel Gea González, Mexico

## Abstract

*Lycium barbarum* is used both as a food additive and as a medicinal herb in many countries, and *L. barbarum* polysaccharides (LBPs), a major cell component, are reported to have a wide range of beneficial effects including neuroprotection, anti-aging and anticancer properties, and immune modulation. The effects of LBPs on neuronal function, neurogenesis, and drug-induced learning and memory deficits have not been assessed. We report the therapeutic effects of LBPs on learning and memory and neurogenesis in scopolamine (SCO)-treated rats. LBPs were administered via gastric perfusion for 2 weeks before the onset of subcutaneous SCO treatment for a further 4 weeks. As expected, SCO impaired performance in novel object and object location recognition tasks, and Morris water maze. However, dual SCO- and LBP-treated rats spent significantly more time exploring the novel object or location in the recognition tasks and had significant shorter escape latency in the water maze. SCO administration led to a decrease in Ki67- or DCX-immunoreactive cells in the dentate gyrus and damage of dendritic development of the new neurons; LBP prevented these SCO-induced reductions in cell proliferation and neuroblast differentiation. LBP also protected SCO-induced loss of neuronal processes in DCX-immunoreactive neurons. Biochemical investigation indicated that LBP decreased the SCO-induced oxidative stress in hippocampus and reversed the ratio Bax/Bcl-2 that exhibited increase after SCO treatment. However, decrease of BDNF and increase of AChE induced by SCO showed no response to LBP administration. These results suggest that LBPs can prevent SCO-induced cognitive and memory deficits and reductions in cell proliferation and neuroblast differentiation. Suppression of oxidative stress and apoptosis may be involved in the above effects of LBPs that may be a promising candidate to restore memory functions and neurogenesis.

## Introduction

Wolfberry, the fruit of *L. barbarum*, is produced mainly in Ningxia, China, and *L. barbarum* have been used as a food additive as well as a medicinal herb in many Asian countries for more than 1000 years [1], [2]. The major active ingredients are thought to be the *L. barbarum* polysaccharides (LBPs), scopoletin and 2-*O*-β-D-glucopyranosyl-L-ascorbic acid (AA-2βG). Studies have suggested that consumption of wolfberry juice may improve the quality of sleep, decrease the level of fatigue and stress [3], and play an important role in preventing and treating various chronic diseases [4], [5], and LBPs are thought to be the major ingredients responsible for these biological activities. LBPs are reported to have anti-aging properties in different models [6], [7] and can suppress oxidative stress [7]–[12]. Several studies focusing on the reproductive system showed that LBP was beneficial to male reproduction by increasing the quality, quantity, and motility of sperm, improving sexual performance, and protecting the testis against toxic insults [13]–[15]. In addition, many other biological activities have been reported, including hypoglycemic [16], [17], hypolipidemic [17], anticancer [18]–[20], and immune effects [19], [21], [22]. In the nervous system, LBPs can protect against neuronal loss induced by β-amyloid peptide [23]–[25], glutamate excitotoxicity [26], and other neurotoxic insults [10], [27]. LBPs are also reported to protect retinal ganglion cells in an experimental model of glaucoma [28]–[31]. Lau *et al.* [15] provided evidence that LBPs could enhance neurogenesis in the hippocampus and subventricular zone. Because LBPs increase hippocampal neurogenesis, LBPs may also regulate learning and memory. However, no studies to date have investigated the effects of LBPs in drug-induced amnesia. In the present study we employed a SCO-induced model of learning and memory impairments to investigate potential anti-amnestic properties of LBPs and their effects on neurogenesis in the brain.

## Materials and Methods

### 1. Animals

Adult male Sprague–Dawley rats (200–220 g) were purchased from the experimental animal center of Nantong University. Animals were housed in a controlled temperature environment (23±2°C), humidity (55%), on a 12 h:12 h light: dark cycle in an approved facility with free access to food and water. All animal experiments were conducted according to approved protocols in the United States National Institutes of Health Guide for the Care and Use of Laboratory Animals. All efforts were made to minimize the number and suffering of animals used in this study.

### 2. Surgery and Drug Treatment

Rats were divided into three groups: the vehicle/saline (veh/sal) group received distilled water and subcutaneous saline; the vehicle/SCO (veh/SCO) group was given distilled water and SCO; the LBP/SCO group received LBPs and SCO. The freeze-dried powder of LBP from Ningxia Rubygoji Ltd is freshly dissolved in distilled water before use. Animals were administered LBP (0.2 or 1 mg/kg body weight per day) or equal volume of water intragastrically for 14 days before SCO treatment. After this period, animals were anesthesized using chlorpent (2 ml/kg) and a 0.5 cm incision was made on the abdominal skin. Alzet osmotic pumps (Durect Co., Cupertino, CA) containing 2 ml saline, or 440 mg/ml of SCO solution were subcutaneously embedded in abdominal wall as previously described [32], [33]. SCO release (0.25 µl/h) was maintained for 28 days and administration of LBPs was continued as before throughout SCO treatment. These schedules were adopted because doublecortin (DCX), a marker of neurogenesis, is exclusively expressed in immature neurons from 1–28 days of cell age [34].

### 3. Novel Object Recognition (NOR) and Object Location Recognition (OLR) Memory Tasks

**Figure 1:**
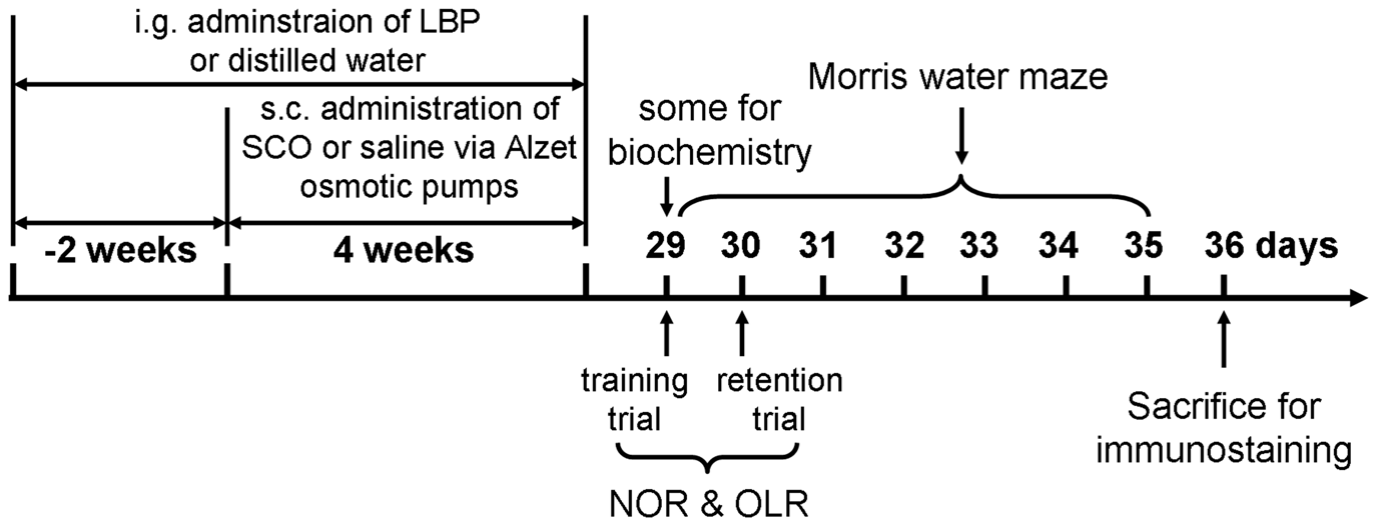
Schedule of the projects. LBPs were administered each day by the intragastric (i.g.) route for 2 weeks, when SCO administration was commenced by a subcutaneous (s.c.) osmotic minipump. Administration of LBP and SCO continued for a further 4 weeks. Behavior tests were performed immediately 1 day after SCO/LBP treatment. Some of animals were sacrificed immediately for biochemical analysis after drug treatment. Twenty-four hours after behavioral testing the rest of animals were sacrificed for immunohistochemical analysis.

As indicated in Fig. 1, after completion of LBP/SCO treatment, rats were caged in an open field arena (65×45×65 cm) in a sound-attenuated room with dim lighting. The NOR and OLR tasks were conducted as described by Han *et al.* (2012) and Okamura *et al.* (2011). Objects were cleaned thoroughly between trials to ensure that olfactory cues were absent. The general procedure consisted of two sessions: a training trial and a retention phase. For the NOR task two different type of objects were employed that were consistent in height and volume but were different in shape and appearance. During habituation, the animals were allowed to explore an empty arena. In the training phase 24 h after habituation, the animals are exposed to the familiar arena with two identical objects placed in opposite sides of home cage at equal distance. In the test session, a familiar object from the sample trial and a novel object were placed in the same locations as in the training phase. The time spent on exploring each object was recorded. Exploration was defined as sniffing or touching the object with the nose and/or forepaws. Sitting on or turning around the object was not considered as exploratory behavior. The time spent sniffing or touching each object was recorded by an observer blind to treatments. A discrimination index (DI) in the test phase was calculated as the percentage of time spent exploring the novel object over the total time spent exploring both objects.

For the OLR task, in the sampling phase two identical objects were placed in different corners of the home cage. The sample trial ended when mouse had explored the two identical objects for a total of 20 s. In the test session, one of the objects was moved to a novel corner (randomly chosen). The retention (test) phase was for 5 min. Object exploration was defined as above. The DI in the test phase was calculated as the percentage of time spent exploring the object in novel location over the total time spent exploring both objects.

### 4. Morris Watermaze

The Morris watermaze test was carried out as decribed by Bromley-Brits et al. [35] after drug administration as indicated in Fig. 1. The apparatus comprised circular tank (1.5 m diameter, 60 cm depth) containing water (27±1°C) to a depth of 40 cm. For the task, rats learned to locate a submerged circular platform (10 cm diameter) at a fixed location over 1 day (5 trials). Various extra-maze cues (posters, door, and computer) were held constant. One day before training, rats were given a pretraining trial for 2 min in the absence of the platform to acclimatize to the situation. On the day of training, rats learned to escape from the water by finding the hidden platform in the central quadrant of the tank. The probe trials were conducted on the last day by removing the platform. In each trial, the animal was placed in the water facing the wall at one of three designated start-points. Animals that failed to locate the platform within 120 s were guided to the platform by an investigator and kept there for 15 s. Escape latency (time to find the platform), swim distance and speed in training trials and time spent in quadrants in probe trial were measured using an auto-tracking system. The average over four tests per day was used for statistical analysis.

### 5. Immunostaining

As indicated in Fig. 1, after behavior tests the rats were sacrificed and perfused with 0.9% NaCl and 4% paraformaldehyde (PFA) in 0.1 M phosphate buffer (PB). Coronal sections (20 µm) through the hippocampus were prepared using a cryostat (Leica CM1900, Germany) and sections were collected into six-well plates containing PBS. Therefore, we got 6 sets of hippocampal sections for immunostaining. For immunofluorescence analysis, sections were first blocked in 10% goat serum in PBST (0.01 M PB containing 0.05% v/v Tween 20) for 1 h at room temperature (RT), and then treated with mouse monoclonal anti-Ki67 (1∶200), anti-calretinin and guineapig anti-doublecortin (anti-DCX, 1∶500) antibodies (Millipore, USA) overnight at 4°C followed by incubation with Alex Fluor 568-conjugated goat anti-rabbit or 488-conjugated goat anti-mouse or guinea pig IgG, respectively. For immunohistochemistry, sections were sequentially treated with 0.3% H_2_O_2_ in PBS for 30 min and 10% goat serum in PBST for 30 min. The sections were then incubated with rabbit anti-DCX (1∶500, Millipore, USA) overnight at room temperature and subsequently exposed to biotinylated goat anti-rabbit IgG (1∶200) and streptavidin peroxidase complex (1∶200). Sections were visualized by a reaction to 3,3′-diaminobenzidine tetrachloride (DAB, Sigma, St. Louis, MO) in 0.1 M Tris-HCl buffer (pH 7.2) followed by dyhydration and mounting on gelatin-coated slides. The positive cells were counted in a series of sections crossing hippocampus representing the total number in the whole hippocampus.

### 6. Western Blot

Hippocampi were dissected from animal after the end of LBP and SCO administration. Protein was extracted using a mammalian Protein Extraction Reagent (Pierce, Rockford, IL, USA) according to the manufacturer’s instructions and separated on 10% polyacrylamide gels in the presence of sodium dodecyl sulfate (SDS). Membranes after transferring were subsequently blocked with 5% non-fat milk in TBS and incubated with mouse monoclonal anti-IGF-1 (1∶1,000, Sigma, St Louis, MO), rabbit polyclonal anti-BDNF (1∶200), -Bax (1∶500), -Bcl-2 (1∶500, Santa Cruz, CA, USA) and mouse monoclonal anti-GAPDH (1∶10,000, Sigma, St Louis, MO, USA) overnight at 4°C. Membranes were developed by incubation with horseradish peroxidase-conjugated goat anti-mouse or rabbit IgG (1∶3,000, Pierce, Rockford, IL, USA) for 2 h at room temperature. After washing, the complexes were visualized by enhanced chemiluminescence (Santa Cruz, CA, USA) and exposed to X-ray film (Kodak, Rochester, NY, USA). The intensity of each band was quantified using the Shine-tech Image System (Shanghai, CHN).

### 7. Biochemical Parameter Assay

As indicated in Fig. 1, after SCO and LBP treatment some rats were anesthetized and decapitated. The hippocampus were rapidly dissected and homogenized in ice-cold PBS to make 10% tissue homogenate that was followed with centrifuge at 3000 rpm for 15 min. The supernatant was used for determination of superoxide dismutase (SOD), glutathione peroxidase (GPx), glutathione (GSH), malondialdehyde (MDA) levels, and acetylcholinesterase (AChE). A BCA protein assay kit (Sigma, Germany) was used for estimation of total protein as described by Smith et al. (1985).

The activity of SOD was assayed as described by Winterbourn et al. [36] by monitoring its ability to inhibit the photochemical reduction of nitroblue tetrazolium (NBT). 200 ul supernatant was used for each 1.5 ml reaction mixture containing 100 mM Tris/HCl (pH 7.8), 75 mM NBT, 2 µM riboflavin, 6 mM EDTA. Monitoring the increase in absorbance at 560 nm followed the production of blue formazan. The enzyme activity is expressed as units/mg protein.

GPx activity and GSH were analyzed by using EnzyChrom™ Glutathione Peroxidase Assay Kit and QuantiChrom™ Glutathione Assay Kit (BioAssay systems, CA, USA) as manual protocols respectively. The absorbance was read in a spectrophotometer at 340 nm for GPx activity assay and 412 nm for GSH assay. The GPx enzyme activity was expressed as units/mg protein, while GSH levels were expressed as nmol/mg protein.

MDA was spectrophotometrically measured by using the thiobarbituric acid assay with the QuantiChrom™ TBARS Assay Kit (BioAssay systems, CA, USA). The final reactions were read at 535 nm. A calibration curve was constructed using standard MDA and the results were expressed as nmol/mg protein.

The activities of enzymes AChE were quantified by using the Acetylcholinesterase (T-CHE) Detection Kit (Nanjing Jiancheng Bioengineering Institute, Nanjing, China), according to the manufacturer’s instructions. The absorbance was read in a spectrophotometer at 595 nm.

### 8. Statistical Analysis

Statistical analyses were carried out using GraphPad software (GraphPad Prism 5.0, San Diego, CA, USA). All data are expressed as means ± S.E.M. Statistical significances of inter-group differences employed one-way analysis of variance (ANOVA) followed by Tukey’s *post hoc* multiple comparison test. *P* values of less than 0.05 were considered statistically significant. Each experiment included at least three replicates per condition.

## Results

### 1. NOR and OLR Task

**Figure 2:**
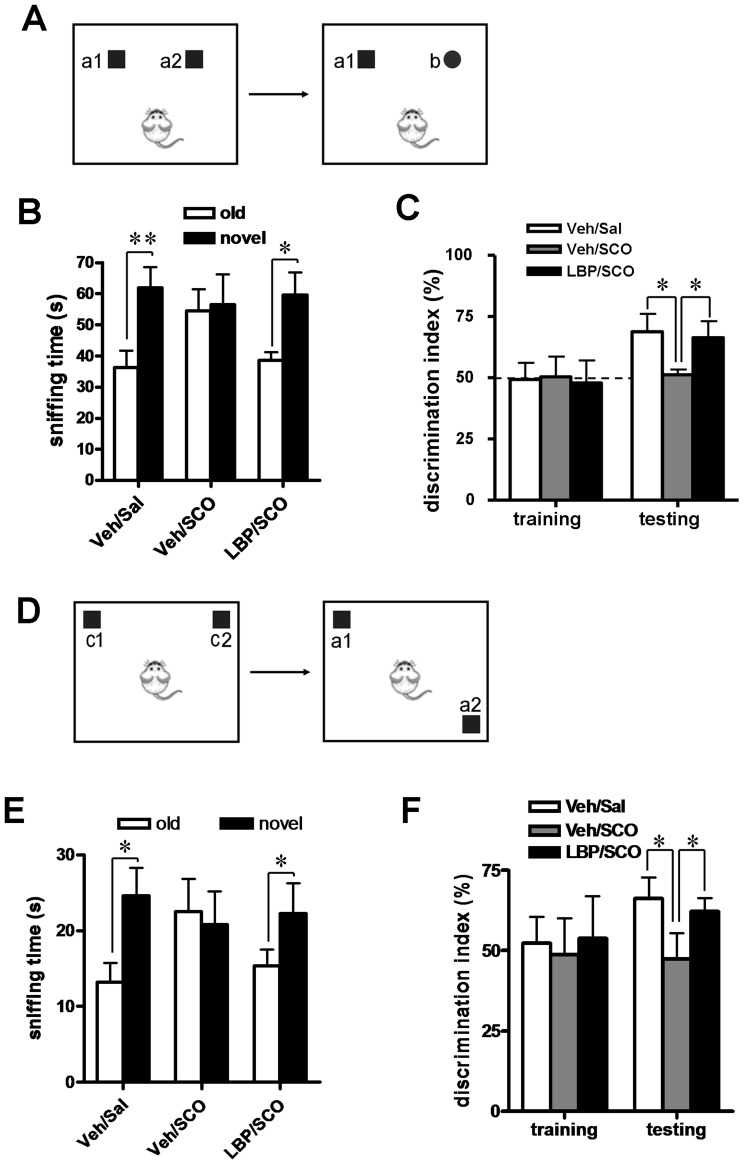
LBPs protect against SCO-induced impairments of working memory. (**A**) Diagram of the novel object recognition (NOR) task. Left (training phase), animals are exposed to two identical objects (a1 and a2). Right (test phase), animal are exposed to two different objects, a familiar (a1) object from the training phase and a new object (b) not seen before. (**B**) The total exploration time in two phases was recorded. Control (vehicle/saline) rats spent more time on exploring the novel object whereas vehicle/SCO animals explored the novel and familiar objects equally. LBP treatment (LBP/SCO group) restored the preference for the novel objects. (**C**) Discrimination indices in the test phase were calculated as the percentage of time spent exploring the novel object over the total time spent exploring both objects. The dashed line indicates the 50% (chance) level. (**D**) The object location recognition (OLR) task. Left (training phase), animals are exposed to two identical objects (c1 and c2). Right (test phase), animals are exposed to two same objects. One object (c1) is at the same location, but the other (c2) is relocated versus the training phase. (**E**) The total time exploring the objects in familiar and novel locations was recorded. Control rats spent more time exploring the relocated object whereas rats treated with SCO (vehicle/SCO group) showed no preference for the object at the new location. LBP treatment (LPB/SCO group) restored the preference for the novel location. (**F**) Discrimination indices in the test phase were calculated as the percentage of time spent exploring the new location over the total time spent exploring both locations. The dashed line indicates the 50% chance level. Values are means ± SEM (Vehicle/saline, *n* = 12; vehicle/SCO, *n* = 10; LBP/SCO, *n* = 11); *,*P*<0.05; **,*P*<0.01.

Behavioral impairments in LBP/SCO-treated and control animals were evaluated using the NOR test to evaluate the ability of rats to discriminate novelty in a complex setting of different objects (Fig. 2A). In the training phase all animals showed the similar discrimination index (DI) for recognition of the two familiar objects (Fig. 2C). In the trial phase the control rats receiving only vehicle spent more time exploring the novel object (60.5±22.8 s) than the familiar one (35.8±17.2 s, *P*<0.01, two-tailed t-test, Fig. 2B). Consistent with the results of previous studies [37], SCO-treated rats failed to discriminate the novel object and explored the novel and familiar objects equally (Fig. 2B) (DI = ∼50%) (Fig. 2C). By contrast, SCO-treated animals receiving LBPs displayed a marked increase in the time exploring the novel versus the familiar objects, and the DI increased from 51.4±7.5% to 65.6±18.6% (*P*<0.05, two-tailed t-test, Fig. 2B), similar to the animals in vehicle/saline group (Fig. 2C), indicating near full recovery of novelty discrimination.

To evaluate the ability of rats to discriminate the new location of an object in relation to spatial information, we performed the OLR test (Fig. 2D). The time the animals spent exploring the two objects at the two familiar corners in the training phase was similar irrespective of whether the animals received SCO or vehicle (Fig. 2F). After a 24 h retention interval rats in the control group spent more time exploring the objects at the novel position (Fig. 2E). Administration of SCO eliminated the trend to explore the novel location, and animals spent equal times exploring the objects at the familiar and novel locations (Fig. 2E, F). However, the extent of the deficit was markedly attenuated in SCO-treated animals receiving LBPs, and SCO/LPB animals showed a strong preference to explore objects in the novel location (*P*<0.05, two-tailed t-test, Fig. 2E, F). These results indicate that LBPs treatment attenuates the effects of SCO on the OLR memory task.

### 2. Morris Watermaze

**Figure 3:**
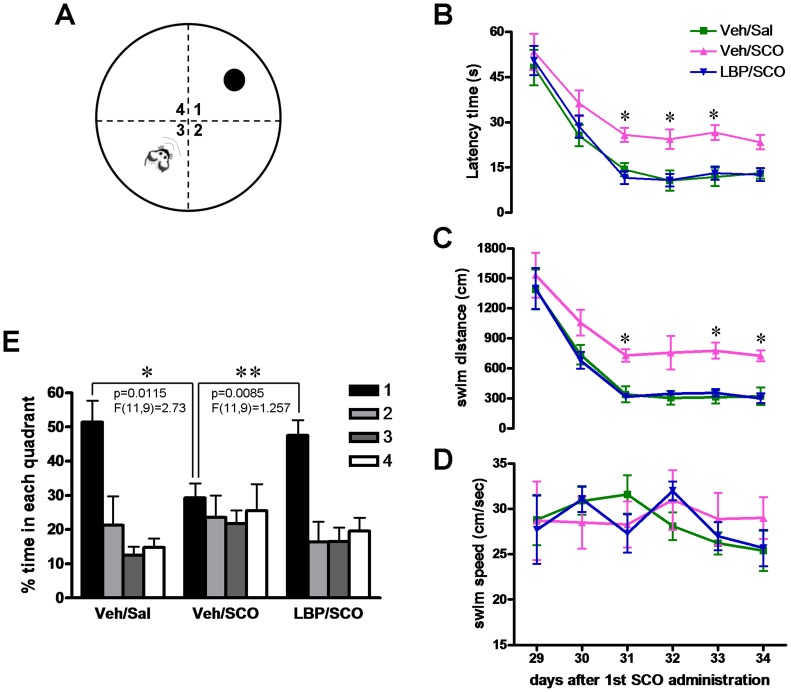
LBP treatment reverses the SCO-induced increase in latency time in the Morris water maze. (**A**) The apparatus for Morris water maze. The black circle in the northeast quadrant represents the location of the hidden platform. Animals were introduced into the southwest quadrant. (**B**) The latency time, swim distance and swim speed to find the submerged platform were recorded over six consecutive days. The animals of three groups have the similar swim speed, but SCO treatment (vehicle/SCO group) increased the latency and swim distance to find the hidden platform whereas LBP administration (LBP/SCO group) restored latency and distance to the levels of the vehicle/saline control group. (**E**) The percentage of time spent in 4 quadrants during the probe trial. The animals in control and LBP/SCO groups spent more time in the target quadrant while SCO treatment decreased time in target quadrant. Values are means ± SEM (Vehicle/saline, *n* = 12; vehicle/SCO, *n* = 10; LBP/SCO, *n* = 11); *,*P*<0.05 versus the vehicle/SCO group.

Spatial memory performance was assessed over successive 6 days using the hidden platform (Fig. 3A) and a probe trial was conducted with no platform on the seventh day. Two-way repeat ANOVA showed significant effects in both group and trial regarding latency time (group: F = 23.37, P<0.0001; trial: F = 47.15, P<0.0001), swim distance (group: F = 24.28, P<0.0001; trial: F = 40.38, P<0.0001) of all three groups, but no significant effect in the factor of group & trial (F_(latency time)_ = 0.5592, P>0.05; F_(swim distance)_ = 0.4242, P>0.05). Administration of SCO significantly increased the latency time (*P*<0.05, Fig. 3B) and swim distance (*P*<0.05, Fig. 3C) to find the hidden platform at six sessions versus controls. However, LBPs treatment substantially improved the SCO-induced memory deficits showing significant decreases in the latency time (*P*<0.05 vs SCO-treated animals, Fig. 3B) and swim distance (*P*<0.05 vs SCO-treated animals, Fig. 3C). The swim speed in SCO treatment groups show mildly lower, but no significance compared other two groups (F = 0.5838, *P*>0.05, Fig. 3D). During the probe trials the percent time spent in each of the four quadrants was calculated and compared within each group using one-way ANOVAs followed with Tukey’s *post hoc* multiple comparison test. The results showed that animals in the control and LPB/SCO-treated groups preferred to spend more time in the target quadrant than other 3 quadrants, while SCO single treatment decreased markedly the time spent in the target quadrant (F(2, 30) = 4.842, P<0.05, Fig. 3E). These results indicated that LBP administration prevent SCO-induced deficits in spatial memory.

### 3. LBP Enhances Neuroprotection and Cell Proliferation in SCO-treated Animals

**Figure 4:**
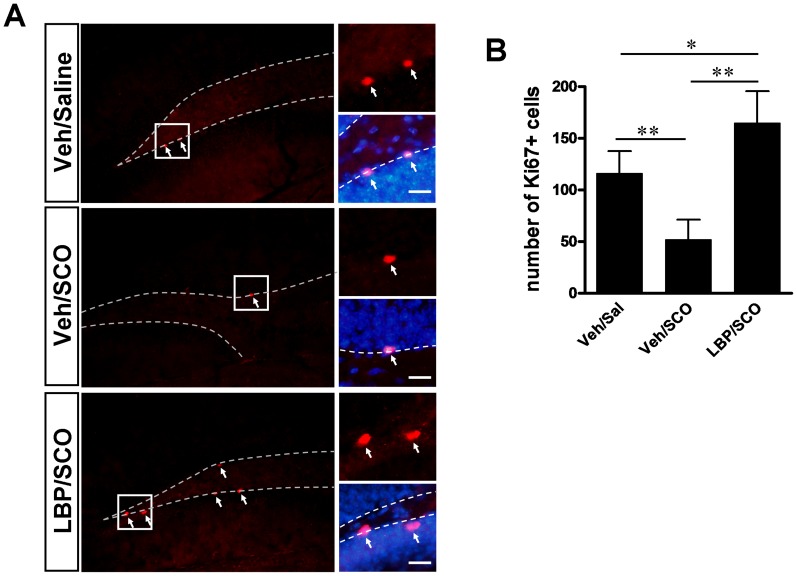
Effect of SCO/LBP treatments on cell proliferation in the hippocampal dentate gyrus (DG). (**A**) Representative immunofluorescence staining for Ki67, a marker of cell proliferation. Sections were subjected to Ki67 antibody after SCO or/and LBP treatment. Arrows indicate Ki67-positive cells in hippocampus. The dashed line is the location of subgranular layer in DG. (**B**) Quantification of Ki67-positive cells in the DG in A. SCO treatment significantly decreased cell proliferation compared to the vehicle/saline group. The LBP/SCO group showed a significantly higher proportion of Ki67-positive cells than the SCO group. Values are means ± SEM (n = 6 animals per groups); *,*P*<0.05; **,*P*<0.01; scale bars, 50 µm.

To assess the effects of LBPs on cell proliferation in the hippocampus we used an antibody against Ki67, a nuclear marker of proliferation, to detect dividing cells in the dentate gyrus. In control animals, Ki67-immunoreactive nuclei were abundantly detected in the subgranular zone of the dentate gyrus (Fig. 4A), with a mean number of Ki67-immunoreactive nuclei per field of 116.3±21.5 (Fig. 4B). SCO treatment was accompanied by a reduction in the number of proliferating cells (52.0±19.4), significantly less than in the control group (Tukey’s *post hoc*, *P*<0.01, Fig. 4A, B). However, numbers of Ki67-immunoreactive nuclei were significantly increased in the LBP/SCO group versus the vehicle/SCO group, with 165.0±30.7 Ki67-immunoreactive nuclei per field (Tukey’s *post hoc*, *P*<0.01, Fig. 4A and 4B). Notably, Ki67-immunoreactive nuclei were more abundant than in the vehicle/saline control group (Tukey’s *post hoc*, *P*<0.05, Fig. 4B). These data demonstrate that SCO significantly reduces cell proliferation in the dentate gyrus but that long-term LBP administration prevents this reduction and, moreover, increased cell proliferation to levels above those observed in control animals.

### 4. LBP Administration Protects Neuroblast Differentiation from SCO Toxicity

**Figure 5:**
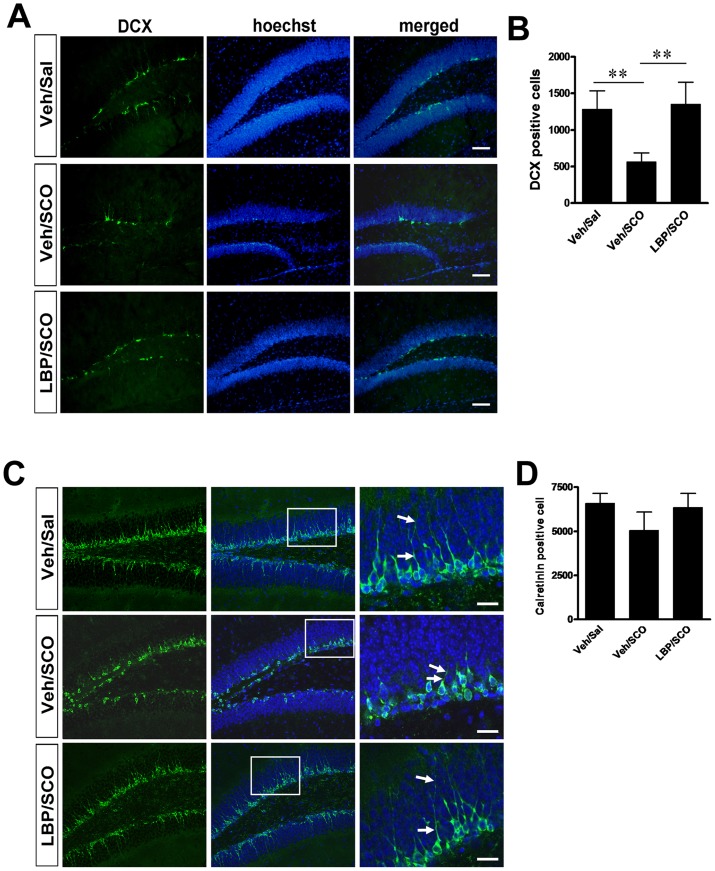
LBP treatment increases neuroblast differentiation in the hippocampal DG. (**A**) DCX immunostaining showed the newborn neurons in the subgranular zone of the DG. DCX-immunoreactive neuroblasts (green) were abundant in the DG in the vehicle/saline group. SCO treatment markedly decreased the number of DCX-positive cells. In the LBP/SCO group, the number of DCX-positive cells was restored. Scale bars, 200 µm. (B) Quantification of DCX-positive cells in DG of the three groups in A. (C) The representative images of Calretinin immunostaining. The length of dendrites is markedly injured by SCO compared with control and LBP treatment groups. Scale bars, 100 µm. (D) Quantification of number of Calretinin positive cells in DG in A. Values are means ± SEM (n = 6 animals per groups); **,*P*<0.01.

Neurogenesis in the hippocampus continues throughout the lifetime of mammals, and newborn DCX-positive neurons can be detected in the subgranular zone into adulthood. These new neurons will sequentially fate to the immature and mature granular neurons of dentate gyrus (DG). We investigated whether LBP/SCO treatment affects numbers of newborn (DCX) or immature (Calretinin) neurons. In control animals, DCX-immunoreactive neuroblasts were abundant along the subgranular zone of the dentate gyrus (Fig. 5A), with 1284±253.5 DCX-positive neuroblasts per field (Fig. 5B). In the vehicle/SCO group, some DCX-immunoreactive neuroblasts were observed (Fig. 5A), but the number (568.5±121.3) was significantly depressed versus control animals (Tukey’s *post hoc*, *P*<0.01, Fig. 5B). However, in the LBP/SCO group the numbers of DCX-immunoreactive neuroblasts (1354±301.4) were significantly increased versus the SCO-treated group (Tukey’s *post hoc*, *P*<0.01, Fig. 5B); the numbers were marginally above those observed in control animals but the difference was not statistically significant (Tukey’s *post hoc*, *P*>0.05, Fig. 5B). Quantification in Figure 5B showed a decrease of Calretinin positive immature granular cells after SCO alone streatment and LBP reversed the SCO-induced decrease although there was no significantly different between three groups. These data demonstrate that whereas SCO treatment significantly reduces neuroblast differentiation in the DG, LBP administration to SCO-treatment animals can prevent this reduction.

### 5. LBP Treatment Enhances Development of Newborn Neurons

**Figure 6:**
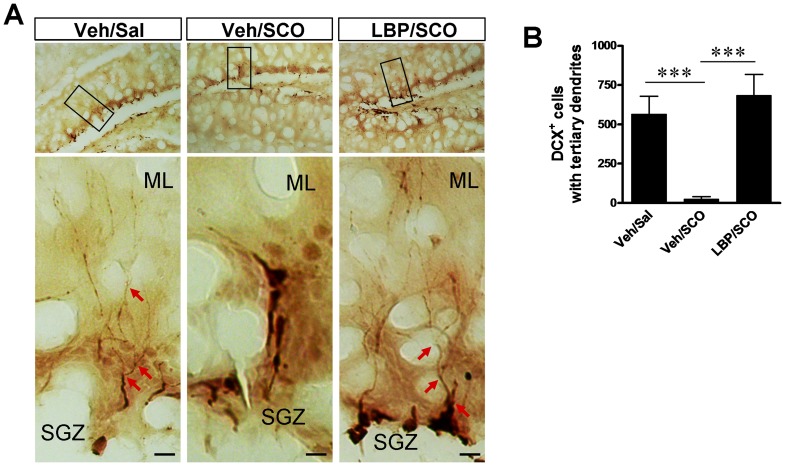
LBP protects the processes of newborn neurons in DG of hippocampus. (**A**) Representative images of doublecortin (DCX)-positive neuroblasts in the subgranular zone of DG. The lower panels are the enlargement of the frames in the upper panels. The arrows indicates the tertiary neurites of DCX positive neurons in DG. In control vehicle/saline and LBP/SCO groups DCX-immunoreactive neuroblasts have well-developed processes extending to the molecular layer of the DG. SCO treatment (vehicle/SCO group) led to significant reduction of tertiary dendrites. (**B**) Quantification of number of the DCX-immunoreactive cells with tertiary dendrites in the three groups. Values are means ± SEM (n = 6 animals per groups); ***,*P*<0.001; scale bars, 50 µm.

The elaboration of dendrites and axons is an important marker of neuronal development. The immature granular cells derived from newborn neurons finally differentiate into mature granular cells, during which their gradually well-developed dendrites with tertiary branches extended into the molecular layer (ML) of DG. Although the number of immature granular cells positive to Calretinin did not show the significant decrease after SCO treatment, the length of dendrites were markedly injured by SCO (Fig. 5C). However, LBP treatment protected the dendrites from damage by SCO (Fig. 5C). Immunohistochemical staining against DCX well displayed the detail of dendrites in DG. As shown in the enlarged image in Fig. 6A, DCX-immunoreactive neuroblasts had typical dendrites extending into molecular layer. In control animals, DCX-immunoreactive neuroblasts with tertiary branches were abundant in the dentate gyrus (Fig. 6A) with 566.2±112.3 per field (Fig. 6B). SCO administration eliminated the majority of tertiary branches; most DCX-positive neurons had only small primary or secondary processes (Fig. 6A), and only 25.4±15.2 DCX-immunoreactive cells with tertiary branches were present, a significant depression versus the control group (Tukey’s *post hoc*, *P*<0.001, Fig. 6B). Notably, long-term LBP administration of LBP markedly reversed SCO-induced tertiary branch loss, and the number of the DCX-positive cells with tertiary branches significantly increased to 685.5±132.6 (Tukey’s *post hoc*, *P*<0.001 vs the vehicle/SCO group, Fig. 6B), a number marginally higher than in the vehicle/saline control group although the difference was not statistically significant. These results indicated that LBP administration can reverse the SCO-induced deficit in the development of the newborn or immature neurons in DG.

### 6. Anti-oxidative Effects of LBP in Hippocampus

**Figure 7:**
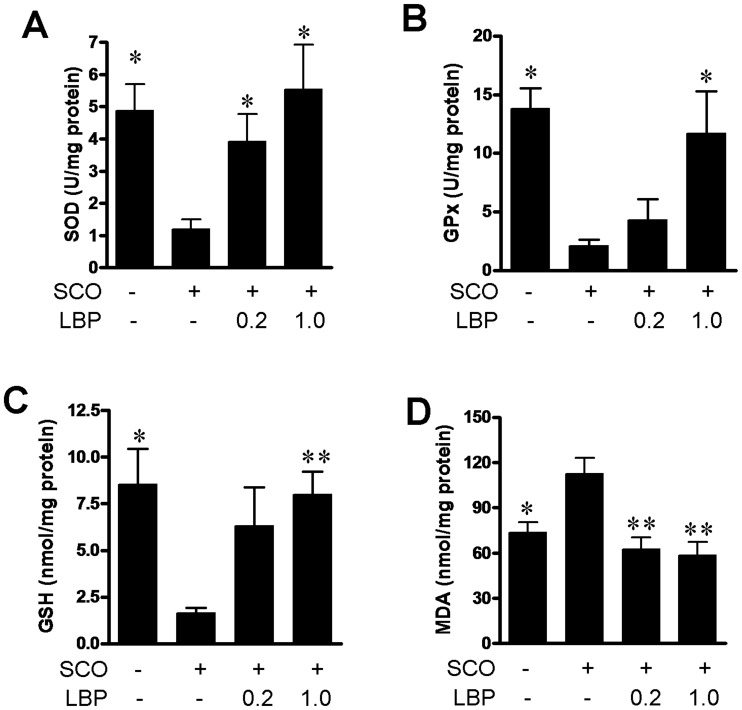
Anti-oxidative Effects of LBP in hippocampus. The SOD (A), GPX (B) specific activities, GSH (C) and MDA (D) levels were determined by using the rat hippocampus homogenates. Values are means ± SEM (n = 4 animals per group), *,*P*<0.05 and **,*P*<0.01 vs. SCO alone-treated group.

To identify the mode of action of LBP on the restoration of memory functions and neurogenesis we started from biochemical analyses of the antioxidant enzymatic activities (SOD, GPX), antioxidant substrate GSH and the lipid peroxidation product MDA in hippocampal homogenates of animals treated with SCO and LBP. As shown in Fig. 7, SCO treatment markedly decreased the levels of SOD (two-tailed t-test, P<0.01), GPx (P<0.05) and GSH (P<0.05) and increase the level of MDA (P<0.01) compared with control. Fig. 7A shows a significant increase of the SOD specific activity (F(3, 12) = 4.317,P<0.05) in SCO-treated animals receiving 0.2 and 1.0 mg/kg LBP administration compared to the SCO-treated group. We also observed a significant increase of the GPx specific activity (F(3, 12) = 6.571, P<0.01) in SCO-treated groups received LBP compared to that of the SCO treated group (Fig. 7B). Post doc test indicated 0.1 mg/kg LBP treatment increase the hippocampal GPx level at 5 folds than the SCO treatment groups (P<0.05). In addition, a significant increase of the GSH release (F(3, 12) = 4.199, P<0.05) (Fig. 7C) after LBP administration compared to the SCO-treated group. Rats in the LBP groups exhibited attenuation in level of MDA, indicated by a significant decrease of MDA level (F(3, 12) = 8.091, P<0.01) (Fig. 7D) compared to the SCO-treated group. Post hoc analyses revealed statistically significant differences between SCO and 0.2 mg/kg LPB+SCO groups (P<0.01), 1.0 mg/kg+SCO groups (P<0.01). The effects of LBP on the above biochemical performances all showed dose dependent. These results demonstrated that the LBP exert a strong antioxidant property in hippocampus.

### 7. Effects of LBP on AChE Levels

**Figure 8:**
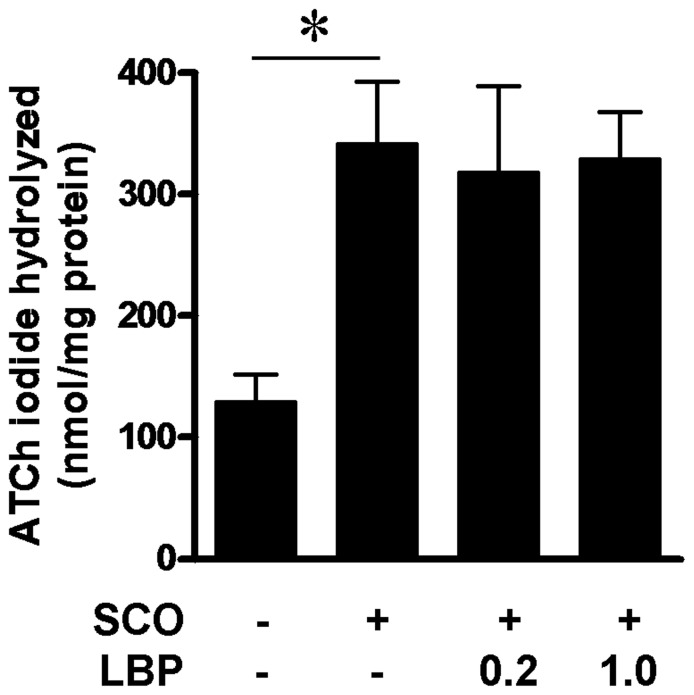
Effects of LBP on the levels of AChE in the hippocampus of the vehicle, scopolamine (SCO), SCO+LBP groups (n = 4 animals per group). The AChE substrate acetylthiocholine in the kits is incubated with hippocampal homogenates. Quantification of the thiocholine produced from the hydrolysis of acetylthiocholine reflects the AChE activities. Values are means ± SEM; *,*P*<0.05 vs. SCO alone-treated group.

The reports [38], [39] have demonstrated that SCO could up-regulate AChE in hippocampus that involved in the SCO-induced learning and memory impairment. So we detected the effects of LBP on AChE in hippocampus. The results showed that the level of acetyl thiocholine (ATCh) iodide hydrolyzed was 130.2 nmol/mg protein in the vehicle group and significantly increased to 342.6 nmol/mg protein after SCO treatment (F (3, 12) = 5.517, P<0.01). However, LBP with different dose did not alter the level of ACTh iodide hydrolyzed compared with SCO-treated groups (Fig. 8). The results that LBP treatment can not suppress the SCO-induced increase of AChE in hippocampus.

### 8. Effects of LBP on Neurotrophic Factors

**Figure 9:**
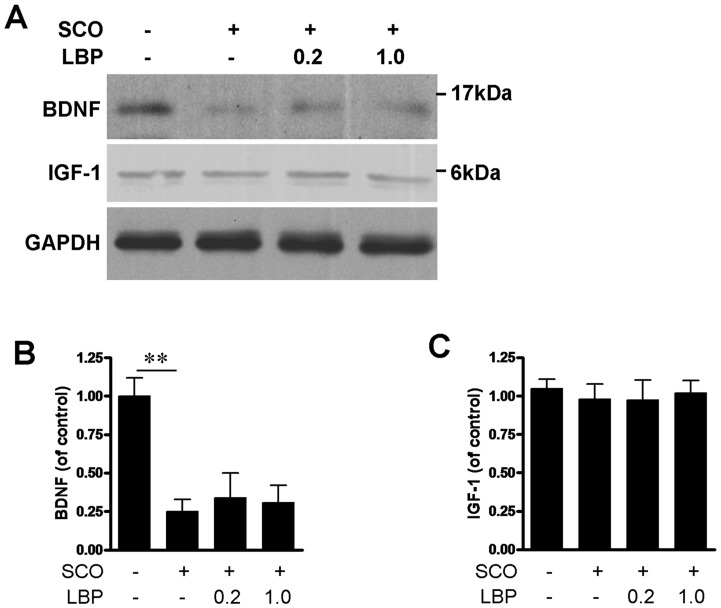
LBP did not alter the expressions of BDNF and IGF-1. (A) The hippocampus homogenates were separated in SDS page and bloted with BDNF, IGF-1 and GAPDH antibodies. GAPDH is the internal standard. (B) Quantification of BDNF in A indicates SCO-decreased BDNF was not reversed by LBP treatment. (C) Quantification of IGF-1 indicates both of SCO and LBP did not influence the level of IGF-1 in hippocampus. Values are means ± SEM (n = 4 in each group). **,*P*<0.01.

The neurotrophic factors BDNF and IGF-1 in hippocampus were evaluated before or after LBP treatment because they all are critical cues involved in hippocampal development and neurogenesis. Consistent with other reports [33], [40], SCO treatment significantly down-regulated BDNF expression in hippocampus (Tukey’s *post hoc*, P<0.01, Fig. 9A and B), but showed no effects on IGF-1 level (Tukey’s *post hoc*, P>0.05, Fig. 9A and C). However, both 0.3 and 1.0 mg/kg LBP did not alternate the levels of BDNF in hippocampus compared with SCO treatment group (F(3, 12) = 0.1429, P = 0.868, one-way ANOVA, Fig. 9B), as well as IGF-1 expression (F(3, 12) = 0.1362, P>0.05, Fig. 9C). It is indicated that LBP can not rescue the decrease of BDNF induced by SCO treatment.

### 9. Effects of LBP on Anti-apoptosis in Hippocampus

**Figure 10:**
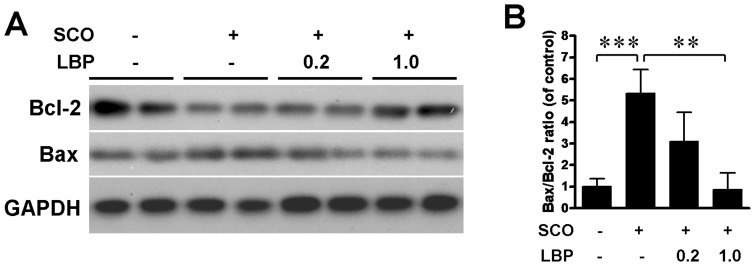
Effects of LBP on the apoptosis in hippocampus. (A) The hippocampus homogenates were separated in SDS page and bloted with Bcl2 and Bax antibodies. Bcl2 level decreases after SCO administration and is reversed by LBP treatment, while Bax shows opposite pattern after SCO or LBP treatment. GAPDH is the internal standard. (B) Quantification of the ratio Bax/Bcl2 in A shows that SCO alone increases the ratio of Bax/Bcl2 which is reversed by LBP treatment. Values are means ± SEM (n = 4 in each group). **,*P*<0.01 and ***,*P*<0.001.

To determinate the anti-apoptosis effects of LBP in hippocampus, the levels of Bcl2 and Bax in hippocampi were evaluated using western blot before or after LBP treatment. Single administration of SCO obviously down-regulated the Bcl2 whereas increased the level of Bax. However, the decrease of Bcl2 and increased Bax all were altered by LBP treatment (Fig. 10A). Quantification in Fig. 10B indicated that the ratio Bax to Bcl2, a well-known index of pro- and anti-apoptosis, significantly increased after treatment with SCO compared with control groups using unpaired t test (P<0.001). Post doc test showed that 0.3 and 1.0 mg/kg LBP significantly suppressed the ratio in a dose dependent manner (P<0.001). The results demonstrated the anti-apoptosis effects of LBP on the SCO-induced toxicity in hippocampus.

## Discussion

The composition of the fruit of Lycium barbarum includes polysaccharides, carotenoids and flavonoids. Polysaccharides (LBP) is the highest content that is approximately 40% by dry mass and represent quantitatively the most important group of substances (as reviewed by Potterat [41]). The LBP used in this study were normally extracted with boiling water, followed by precipitating with ethanol, protein hydrolysis, dialysis, and fractionation with a DEAE-Sepharose CL-6B column as previous description [9], [42]. This method results LBP generally consisting of six monosaccharides (galactose, glucose, rhamnose, arabinose, mannose, and xylose) that mainly attributed to the bioactivity. People always intake Lycium barbarum via drinking commercial fruit juice or boiling soup since it is usually used as a food additive, as well as a medicinal herb. This is a chronic and long-term procedure to enable its bioactivity to function within various organs. A clinical trial conducted by Amagase et al. designed the totally 30-day intake period of Lycium barbarum juice as 30 days [3]. So the LBP in this study was administered for a long period. To coordinate the chronic administration of LBP we chose subcutaneous chronic administration of SCO via osmotic pumps. Although this model is rarely used, the reports and our results suggested that chronic administration of SCO in fact induced not only hippocampal damage [43], [44] but also cognitive and memory deficits (present data).

The hippocampus is an important region of the cholinergic system in the forebrain of humans and other mammals and plays crucial roles in spatial navigation and consolidation of long-term memory. There is continuous regeneration throughout life of new neurons in the hippocampus, and the DG displays neurogenesis into adulthood [45]–[47]. Newly proliferating cells in subgranular zone (SGZ) of the DG migrate into the granule cell layer, mature into new neurons sending axons to the CA3 region to form mossy fibers and projecting dendrites to the outer molecular layer receiving input from entorhinal cortex, and finally form functional synaptic connections with the hippocampal circuitry [48]–[50]. In adults these processes are thought to play a crucial role in the establishment and maintenance of memory traces and spatial navigation [51], [52].

SCO is thought to exert various toxic properties on the nervous system. In this study it exhibited toxicity on the population and dendritic development of the newborn neurons and immature granular cells in DG, which directly results in injury of the hippocampal circuits that may predominantly be responsible for cognitive and memory deficits. The extent to which the hippocampal circuits were destroyed needs further evaluation using electrophysiological meseaures in the following investigation. Inhibition of the muscarinic acetylcholine receptor by SCO also contributes to characteristic cognitive and memory deficits of Alzheimer’s disease (AD) [33], [44], as well as the cholinergic receptor antagonists [43], [53]. Our results together with other reports [38], [39] indicate that AChE activity in hippocampus was increased after SCO treatment, which partially mediated SCO-induced neurogenesis impairment in hippocampus. Oxidative stress is thought to be involved in the pathogenesis of dementia and age-related neurodegenerative disorders, and reactive oxygen species (ROS) are implicated in age-related cognitive decline and AD development [54]–[57]. Oxidative stress is another toxic reactivity induced by acute or chronic SCO treatment. Our study and reports from others [58], [59] showed that SCO treatment significantly promoted oxidative stress, such as decreasing activities of SOD, GPx, catalase (CAT) and increasing MDA levels, which may further promote the programming apoptosis [58], [60], decrease of cell proliferation and loss of dendrites of newborn neurons. In this study, we found that SCO also down-regulated the hippocampal critical factor BDNF expression, which is consistent with the previous reports [44], [61]. These mechanisms, more or less, are responsible for SCO-induced dysfunction of memory and spatial navigation and even the neurogenesis impairment.

The present study demonstrated LBP administration not only enhanced cell proliferation and prevented neuroblast differentiation from SCO toxicity in the DG but also ameliorated the cognitive and memory function. In the light of the close relationship between hippocampus and the learning and spatial navigation, it is deduced that the increased neurogenesis in hippocampus elicited by LBP administration may underlie the improvements in learning and memory tasks. The mechanisms underlying the neuroprotective effects of LBP on cell proliferation and neuroblast differentiation are not yet known. To reveal the mode of action of LBP, we detected a serial biological factors related with SCO or LBP in hippocampus. Although SCO stimulation exerted multiple toxic effects on the hippocampus, LBP treatment only rescued the neurogenesis and neuroblast differentiation and reversed oxidative stress and apoptosis, but did not alter the SCO-induced elevation of AChE activity and decrease of BDNF. The results well implied that the anti-oxidative and anti-apoptotic effects underlie the neuroprotective effects of LBP on the hippocampal structure and functions.

The antioxidant activities of LBPs have been widely studied *in vitro* and *in vivo*. LBPs are reported to be effective scavengers of OH free radicals and superoxide anions, and show concentration-dependent inhibition of DPPH^−^ and ABTS**·** ^+^ free radicals [62]–[64]. LBP treatment has been reported to exert protective effects in the conditions in which oxidative stress is thought to play a central role, including ischemia/reperfusion injury, high-fat diet (HFD) feeding, intense exercise, and aging [10], [65]–[69]. The antioxidant effects of LBP in humans have also been examined by Amagase *et al.* [3]. Fifty Chinese healthy adults aged 55–72 years were investigated in a 30 day randomized, double-blind, placebo-controlled clinical study. LBP treatment significantly increased serum levels of SOD (+8.4%) and GSH-Px (+9.9%), and decreased serum MDA content (–8.7%). These reports combined with our results could suggest that the protective effect of LBP is mainly mediated by changes in antioxidant enzymes and reduced oxidative stress. Oxidative stress directly reults the sequential response apoptosis. Our results indicated that SCO indeed induced apoptotic response in hippocampus, which is consistent with other reports [58], [60]. The ratio Bax/Bcl2, the apoptotic index, was markedly down-regulated by SCO, while LBP administration reversed the ratio. It is reported that Bax-mediated programmed cell death of adult-generated neurons takes place during an early phase of differentiation, which further demonstrated that pro-apoptosis of SCO mediates destroy of the tertiary branches of the newborn neurons and the anti-apoptosis of LBP prevents the injured branches from toxicity. Therefore it is suggested that the protective effect of LBPs against SCO-induced impairments is mediated by inhibition of damage induced by free radicals and the sequential apoptosis after SCO administration.

In conclusion, our results demonstrate that LBP administration can prevent the cognitive and spatial navigation and hippocampal neurogenesis from damage by SCO and the mechanisms underlying the neuroprotective properties of LBPs include the antioxidation and anti-apoptosis. Therefore, there is widespread interest in the use of LBP as an antioxidant to promote hippocampal neurogenesis and neuronal survival in aging or neurodegenerative disorders.
